# Food consumption and nutrient intake among adolescent girls in The Gambia: a cross-sectional study with urban-rural and nutritional status comparisons

**DOI:** 10.1186/s40795-026-01430-0

**Published:** 2026-07-17

**Authors:** Haddy Jallow-Badjan, Tanefa A Apekey, Maria J Maynard

**Affiliations:** 1 Global Ethnicity, Migration and Health Research Group, Leeds Beckett University, Leeds, UK; 2 Sheffield Centre for Health and Related Research, University of Sheffield, Sheffield, UK

**Keywords:** Adolescent, Food Consumption, Nutrient intake, Nutritional Status, Rural Population, Urban Population, Gambia, Cross-sectional Study

## Abstract

**Background:**

Heightened biological and sociocultural vulnerability among adolescent girls places them at increased risk of malnutrition in The Gambia, as elsewhere. This cross-sectional study therefore aimed to explore food consumption and nutrient intakes comparing urban and rural and nutritional status groups, providing novel data for The Gambia.

**Methods:**

Adolescent girls aged 10–19 years from urban or rural communities took part in the study. Participants completed a questionnaire collecting socio-demographic information. Anthropometric measurements were obtained to derive body mass index-for-age and height-for-age z-scores, and nutritional status (stunting, underweight, and overweight). Dietary intake was assessed using two interviewer-administered multiple-pass 24-hour dietary recalls.

**Results:**

A total of 208 adolescent girls participated in the study. The diets of those in urban areas featured frequent consumption of processed cereals, sandwiches, and meat-based stews. By contrast, traditional porridges and fish-based stews predominated in the diets of rural participants. Overall, diets in both groups were energy-dense, but low in fruit and vegetables (180 g per day, on average), fibre and iron. Excess fat and carbohydrate as a percentage of energy intake featured in the diets of both groups. Despite greater intakes among the urban compared to rural adolescents for vitamin A (222 vs. 164 µg per day), vitamin D (2 vs. 1 µg) and calcium (390 vs. 289 mg), deficiencies were evident for both urban and rural participants. Higher intakes of free sugars among urban adolescents, inadequate vitamin C intakes among rural adolescents, and (in the context of deficient intakes overall) lower calcium intakes among adolescent girls who were stunted compared to those who were not (325 vs. 367 mg; *p* = 0.018), were also notable disparities. White rice provided nearly half of total energy intake, and nearly 40% of daily protein across settings. The predominance of sugar-sweetened beverages, and the importance of traditional sweetened drinks as a source of vitamin C, is also a notable concern.

**Conclusions:**

This study describes similarities and differences in urban and rural diets in The Gambia, with nutritional inequalities evident in both settings. The findings are context-specific and descriptive, but are likely to resonate with other West African contexts and underscore the need for targeted intervention strategies to address malnutrition among adolescent girls.

**Supplementary Information:**

The online version contains supplementary material available at https://doi.org/10.1186/s40795-026-01430-0.

## Background

Adolescent malnutrition is a critical public health concern, with disproportionately high prevalence and consequences in sub-Saharan Africa [SSA] [1]. Ensuring adequate nutrition during adolescence is increasingly recognised as a global health and development priority, central to achieving the Sustainable Development Goals and breaking intergenerational cycles of malnutrition [2, 3]. Adolescence (ages 10–19 years) is a pivotal stage of growth and development during which adequate nutrition is essential for immediate health and for achieving optimal adult and reproductive outcomes [4]. Across the SSA region inadequate dietary intake of fruit and vegetables, animal protein and essential nutrients among adolescents have been reported alongside rising consumption of sugar-sweetened beverages and processed foods [5–7]. Across SSA, this population group are at risk of the triple burden of malnutrition [7] with co-existing micronutrient deficiencies, underweight, and overweight and obesity [1, 8] leading to increased risk of diabetes and cardiovascular diseases later in life [9]. Observational data on adolescent diets in SSA countries therefore provide the foundations for understanding malnutrition. Complex interventions are needed to address this multifaceted health issue [10], and detailed knowledge of food and nutrient intake is vital evidence for informing tailored, nutritional intervention components.

In The Gambia, adolescents comprise about one quarter of the population [11]. Undernutrition among adolescents in The Gambia remains both prevalent and persistent. National data and recent research report prevalence rates of underweight at 9–31% [12–15], and stunting at 27–41%, with rural adolescents carrying the highest burden [13–15]. Anaemia is also widespread, affecting 35–41% of adolescents [12, 15, 16]. Overweight and obesity among adolescents in The Gambia is relatively low at < 10%, but showing an upward trend over recent years, especially among urban populations [12, 15, 17]. Food insecurity, early marriage, and adolescent childbearing exacerbate nutritional vulnerabilities, particularly among girls [15, 18]. Such vulnerability among girls in The Gambia is evident in the excess risk of underweight, overweight and obesity compared to boys [17]. Therefore, gaining understanding of food and nutrient intake among adolescent girls, and examining intakes in relation to recommendations and nutritional status is essential.

Research on the diets of adolescents in The Gambia has employed a range of approaches, involving qualitative studies of food practices, biological assessments, school-based surveys, and analyses of urban-rural disparities [13, 14, 17–19]. Together, these provide multidimensional understanding of adolescent nutrition. However, important evidence gaps remain on comprehensive assessment of food, energy and nutrient intakes in both urban and rural settings. Previous dietary studies among urban [17] and rural [13] adolescent populations have relied primarily on frequency-only food frequency questionnaires without portion size quantification, which cannot provide reliable estimates of actual food consumption or energy intake. As a result, current evidence offers limited understanding of the quantity and nutritional quality of foods consumed by adolescents. Furthermore, although some studies have examined adolescent nutrition in either urban or rural settings, robust direct comparisons of dietary patterns, nutrient intake, and nutritional status among adolescent girls across these contrasting environments remain limited [20]. This is particularly important given the rapid urbanisation and ongoing nutrition transition occurring across many low- and middle-income countries, including The Gambia, which may influence food environments, dietary behaviours, and malnutrition risk differently across settings [18, 21, 22].

Weighed and estimated food intake methods have previously been applied in the development of The Gambia Food Composition Table to estimate portion sizes in rural village settings [23]. The study provides valuable country-specific data on food consumption, portion size estimation, and nutrient composition that informs the dietary assessment methodology in the present paper. However, this work was restricted to a rural population, focused on pregnant and lactating women rather than adolescents, and was conducted over a decade ago, limiting its applicability to contemporary adolescent populations. To our knowledge, no previous study has comprehensively quantified food consumption, energy intake, and nutrient intake among adolescent girls across both urban and rural settings in The Gambia. To address these gaps, the present study provides a comprehensive assessment of food consumption, energy, and nutrient intakes among adolescent girls in both urban and rural settings, and by nutritional status, in The Gambia. By generating contemporary quantitative dietary data and comparing nutritional patterns across contrasting settings, this study contributes new empirical evidence to inform context-specific large observational studies, intervention development, and nutrition policies aimed at reducing adolescent girls’ malnutrition.

Malnutrition among adolescent girls in The Gambia is therefore a significant public health issue and data on diet and nutritional status are needed to understand and improve these outcomes. Therefore, the overall aim of this study was to use data collected via multi-pass 24 h recalls and anthropometric measures to describe food and nutrient intakes, and to consider intakes in relation to nutritional status of adolescents, in rural and urban areas of The Gambia. Objectives were to: (1) Identify commonly consumed dishes and other prepared foods; (2) Calculate energy and nutrient intake and compare these by settings, nutritional status (underweight, overweight and stunting) and with recommendations; and (3) Describe the energy and nutrient contribution of selected food groups. Our intention was to explore the phenomena of interest in this descriptive study rather than test hypotheses, therefore generating data to inform analytical studies.

## Materials and methods

### Study design and setting

This quantitative, cross-sectional study was part of a larger survey on adolescent nutrition conducted in Brikama and Mansakonko Local Government Areas (LGAs) of The Gambia, chosen to capture urban-rural contrasts [16]. One urban community in Brikama and two rural villages in Mansakonko were selected to reflect differences in infrastructure, livelihoods, and food access. Brikama is more urban and ethnically diverse, and Mansakonko predominantly rural and Mandinka [15]. In relation to the national context, together Brikama LGA, the most populous LGA, and Mansakonko LGA account for approximately 51% of the population in The Gambia [24]. Household sizes in both LGAs (7.7–8.3 persons) are comparable to the national average of 8.0 persons [24]. Engagement in farming observed in Mansakonko (79% of households) is similar to that reported across other rural LGAs (76–92%). More than one-third of the country’s buildings are located in Brikama LGA [24]. By contrast, less than 5% of buildings are in Mansakonko, comparable to other rural LGAs such as Kuntaur (6%) and Janjanbureh (8%) LGAs [24]. Although not intended to be representative, these chosen settings were appropriate for urban-rural comparisons.

The study builds on a prior mixed-methods exploratory phase [19] that informed the cultural context, feasibility of recruitment, and development of the socio-demographic questionnaire used in the present study. The current study is reported in accordance with the Strengthening the Reporting of Observational Studies in Epidemiology- Nutritional Epidemiology (STROBE-NUT) guidelines [25] (see Supplementary File S1 for the completed checklist). The study was led by the first author (HJB) under supervision of the co-authors (MJM, TAA), with data collected by a team of trained field workers managed by HJB.

Ethical approval was obtained from the Research Ethics Committee, School of Health, Leeds Beckett University (Ref: 73304), and The Gambia Government/Medical Research Council Unit The Gambia at the London School of Hygiene & Tropical Medicine (MRCG) Joint Ethics Committee (Ref: R020021). In addition, permission was secured from local authorities, including village chiefs (*Alkalos*), as well as village and compound heads within the study areas. Written informed consent was obtained from parents or guardians of participating adolescent girls aged under 18 years, and from those aged 18 years and above, alongside assent from all adolescents, through the signing of consent and assent forms. The study was conducted in accordance with the principles of the Declaration of Helsinki.

### Sampling and recruitment

Participants were female adolescents aged 10–19 years old. Purposive sampling was used based on the characteristics age, gender, and residence in the rural or urban areas. Sample size was calculated using the standard single population proportion formula: n= Z^2^P(1-P)/d^2^ where $\:n\:$is the required sample size, Z corresponds to the 95% confidence level (1.96), P is the assumed prevalence (50%, conservatively chosen in the absence of directly applicable prevalence estimates), and d is the acceptable margin of error (7.5%). A design effect of 1 was assumed. Clustering is a likely factor in this setting and with the community engagement approach but was not incorporated into the sampling design to ensure a feasible sample size for depth of enquiry. As a cross-sectional study primarily designed to describe dietary and nutritional outcomes, sample size determination was based on precision rather than formal hypothesis-testing power calculations. The calculated minimum sample size was then adjusted for anticipated non-response using: n_adjusted_ = n/1-q where q represents the anticipated non-response/dropout proportion (a 10% adjustment was applied). This resulted in a final target sample size of approximately 192 adolescent girls (96 per setting). This sample size is also broadly consistent with survey sample size guidance for populations of similar size [26].

Recruitment was facilitated through community focal persons and engagement with parents to raise awareness about the study, and provision of small incentives, such as school stationary, as previously described [19]. All participating adolescent girls were recruited from June to July 2021, with faster enrolment in rural settings due to well-established local structures such as village development committees and mothers’ clubs, smaller populations, and greater receptivity to researchers compared with the urban site [19].

### Measures

#### Socio-demographic questionnaire and nutritional status measures

Participants completed a socio-demographic and health questionnaire, designed and refined as part of the previous study [19]. Questions relevant to the current study included age, urban or rural residence, country of birth, ethnicity (tribal affiliation) and ever attended school. As previously described [19], body mass index (BMI)-for-age z-scores and height-for-age z-scores were calculated from weight and height measures with protocols informed by WHO guidance [27, 28].

#### Dietary assessment

Two multiple-pass 24-hour dietary recalls were conducted to assess dietary intake, owing to their validated nature, minimal respondent burden, and widespread use in low-resource settings [29]. The multi-pass interviewer-assessed 24 h recall involves a staged approach thought to be better tailored to human cognition than the chronological approach [30]. The five stages are: (1) ‘Quick list’, to collect a list of foods and beverages consumed during the previous day (i.e., over past 24 h) including snacks; (2) ‘Forgotten foods’, to probe for foods forgotten during the quick list; (3) ‘Time and occasion’, to record time and eating occasion for each food; (4) ‘Detail cycle’, to record detailed description, amount, use of condiments, cooking methods, brands of foods if purchased, and other information (e.g. whether the day’s food intake was commensurate with other days and any dietary restrictions or health condition that may influence intake level) as a review of the 24-hour day; and (5) ‘Final probe’, to probe anything else consumed. Participants were asked to estimate portion sizes by using common household items such as local plastic drinking cups and wooden ladles used for porridges [31]. At the end of each recall session, the interviewer checked the record for completeness and accuracy. For recipes of composite dishes, participants were asked to list all the ingredients, amounts and preparation methods. Where unsure, they were encouraged to ask a household member, usually a parent/guardian, older sibling, or other family member engaged in the cooking process, for this information.

Assessing intakes where communal eating and eating with the hand are common practices (as is the case in The Gambia) can be particularly challenging. Insights from our previous study have been invaluable in understanding local food practices [19]. These included communal eating segregated by age and gender mainly in rural settings, restrictions on eating certain foods (eggs, bitter tomatoes), and the prioritisation of large portions for the men in households [19]. These insights were combined with published in-country data [23] to develop context-relevant quantification of portion size estimated from the number of spoon or handfuls of each dish consumed. For example, participants were asked whether they used a spoon or their hand for solid foods like rice or millet-based couscous and porridges. A 10 ml spoonful of boiled rice or porridge was equated to 15 g and 10 g respectively, and a handful of boiled rice with or without sauce was estimated as weighing 60 g [23]. The total amount was calculated for each meal. Recalls were handwritten on an open-ended record sheet detailing the description of food or beverage, ingredients, preparation method, estimate of amount consumed (portion size), and time and place consumed.

### Data collection procedures

Data collection was carried out in July and August 2021, following consent and assent procedures. In the rural areas data collection took place in a school and the *Alkalo’s* compound. In the urban setting, data were collected in a school, a *Dara* (an informal Islamic school), and a community focal person’s compound. Each day of the data collection ran from 10am to 7pm to allow flexibility in respondent attendance. All data collection was conducted by the first author (HJB) together with a team of eight fieldworkers. To minimise observer-related variation, all interviewers received standardised training in dietary assessment procedures and use of household measures for portion-size estimation [19]. Data collection followed a standardised protocol, and completed dietary recalls were regularly reviewed among the research team for consistency and quality. Questionnaires were completed with support from the fieldworkers, and the anthropometric measures were then taken. Subsequently, the first dietary recall was conducted in person at the data collection site. Approximately one week later, the second recall was administered by telephone. To capture day-to-day variation in intake, one recall was conducted on a weekday and the other on a weekend day. Evidence suggests that telephone-administered, multiple-pass dietary recalls provide similar reported energy intake and diet quality compared to in-person assessments [31, 32].

### Dietary data management and analysis

All dietary data were entered into Nutritics food and nutrition analysis software (version 5.87, 2019) [33]. For the food level analysis, items were coded within the software, the data exported into MS Excel and summed to calculate daily intake of the most popularly consumed (by at least 15 participants in either setting) individual dishes and their portion sizes, as well as major food groups (e.g. total fruit and vegetable consumption). As part of quality assurance, a subsample of 20 dietary recalls (approximately 10% of the total sample) was reviewed to assess potential differences in reporting depth, quantity of food items reported, and portion size estimation between the face-to-face and telephone-administered recalls. This assessment indicated comparable reporting across both methods, with a similar number of food items reported in the face-to-face and telephone recalls. Portion size estimates were also broadly consistent between the two modes of administration. For example, when participants described meal portions using familiar household measures such as handfuls, spoons, or ladles, the reported quantities were similar across both recall methods.

The Nutritics software holds composition data for over 750,000 foods through a compilation of databases from a range of countries including the UK, other European countries, north African and Middle Eastern countries, and the United States [33]. Across the databases the composition of foods and ingredients such as rice, vegetables, and spices are relevant to the Gambian context, with the assumption that the energy and nutrient composition of these ingredients are comparable to those as consumed in The Gambia. Additional composition data for specific, local ingredients not already available in the range of databases within the system were manually added to the software using the Food Composition tables for The Gambia [24] and West Africa [35]. Recipes were created for all composite dishes reported. Energy and nutrient analysis per 100 g of each recipe was therefore based on a combination of Gambian or West African data, and the wider data, as described. For example, analysis of a fish stew recipe would include the composition of local fish (e.g. *kobo* from Food Composition tables for The Gambia [23], and cherry tomatoes from the Nutritics system. Intakes of estimated daily energy (kcals/ MJs) and key macro and micronutrients (e.g. protein, fat, carbohydrate, fibre, calcium, iron) were calculated.

### Statistical analysis

Complete case analysis was conducted (missing data were *≤* 4% for each data item). Food, energy and nutrient intakes, and age were continuous variables; all other socio-demographic variables were defined as categorical variables. The normality of continuous variables was tested using the Kolmogorov-Smirnov test. Descriptive statistics were calculated according to variable type, using means and standard deviations (SDs) for continuous, normally distributed data, median and interquartile range (IQR) for skewed data, and counts and percentages for categorical data. T-tests (normally distributed data), Mann-Whitney U, Kruskal–Wallis, and Chi-squared/ Fishers Exact tests (skewed data/ proportions) were used to identify statistically different patterns between groups, such as urban vs. rural participants (*p* < 0.05), with sensitivity analysis conducted stratifying by early (age 10–14 years) and later (age 15–19 years) adolescence. However, due to the sample size and sampling limitations (non-probability sampling; no accounting for clustering), interpretation regarding statistical inference should be cautious. As a dietary data quality check, under- and over-reporting of energy intake was determined by calculating the ratio of energy intake to estimated basal metabolic rate from published data (EIrep: BMRest) for each participant [34]. Lower and upper cut-offs for acceptable reporting were calculated based on equations from Black [35]. Median EIrep: BMRest for the sample was 1.73 (IQR = 1.73–2.06). The calculated lower cut-off (EIrep: BMRest = ≤ 0.87) and upper cut-off (EIrep: BMRest = ≥ 2.25) were used to classify each participant as under (below lower cut-off) acceptable (within lower and upper cut-offs) and over (above upper cut-offs) reporters. Sensitivity analyses, including acceptable reporters only, compared with the full sample, were conducted where necessary to examine the potential impact of misreporting. All statistical analyses were conducted using IBM SPSS Statistics (Version 29).

## Results

### Socio-demographic characteristics and nutritional status

#### Socio-demographic characteristics

Sample characteristics are summarised in Table 1. A total of 208 adolescent girls (*n* = 124 from the urban setting; *n* = 84 from the rural setting) took part in the study, exceeding the target sample by 16. Two 24 h recalls were completed by 98% of participants. The five participants that did not complete the second dietary recall were not accessible when contact was attempted during the follow up period and thus were not included in the dietary analysis. Most participants were categorised as acceptable reporters (81%), with negligible under-reporting detected (1%). Almost all of participants were born in The Gambia (98%) and were attending school (99%). The rural participants were predominantly from the Mandinka tribe, with more diversity of ethnic groups in the urban setting, including Wolof, Jola and others. All of the adolescents in the rural villages identified as Muslims. In addition to the majority being Muslim among urban participants, a small number identified as Christian.

### Nutritional status variables

In line with previous reports [12, 15], stunting was more common in participants from the rural areas compared to urban settings (49% vs. 18%; *p* < 0.001). Urban participants had higher mean BMI than those from the rural areas (20 vs. 18 kg/m^2^; *p* < 0.001). There were no notable differences in weight status between settings; however, also consistent with previous data [12, 15], 31% of participants across settings were classed as underweight.

**Table 1:** Socio-demographic characteristics and nutritional status, by setting

		*N* (%)^a,b,c^		*P*-value^d^
	All	Urban	Rural	
Socio-demographic characteristics				
Total sample size	208 (100)	124 (60)	84 (40)	**0.006**
Country of birth^c,e^ Gambia	203 (98)	119 (97)	84 (100)	0.095
Age, years, mean (SD)	15 (3)	16 (3)	14 (3)	**< 0.001**
Ethnic group Mandinka Other	125 (60) 83 (40)	51 (41) 73 (59)	74 (88) 10 (12)	**< 0.001**
Religion				
Muslim Christian	191 (92) 17 (8)	107 (86) 17 (14)	84 (100) N/A	**0.002**
Attended school				
Yes	207 (99)	123 (99)	84 (100)	0.409
Completed 2 × 24 h recalls				
Yes	203 (98)	120 (97)	83 (99)	**-**
Energy intake reporting quality^c,f^				
Acceptable reporters Over reporters	169 (81) 32 (15)	97 (81) 21 (17)	72 (86) 11 (13)	0.387
Nutritional status				
Stunted (based on height for age z score)^g^				
Yes No	64 (31) 144 (69)	23 (18) 101 (82)	41 (49) 43 (51)	**< 0.001**
Body mass index (BMI),				
mean (SD) kg/m^2^	19 (4)	20 (4)	18 (3)	**< 0.001**
Weight status (from BMI for age z-scores)^e,h^				
Underweight	65 (31)	37 (30)	28 (34)	0.187
Normal weight	126 (61)	74 (60)	52 (62)	
Overweight/obese	16 (8)	13 (10)	3 (4)	

^a^Values are N and column percentages unless otherwise stated, ^b^*N* = 208 unless otherwise stated, ^c^Categories within variables where n = < 5 are not reported, ^d^From chi2 or Fisher’s Exact tests, bold type = statistically significant (*p* < 0.05), ^e^*N* = 207, ^f^*N* = 203, ^g^Height for age z score cut offs used to define stunting, WHO growth standards [27], ^h^BMI for age z score cut offs used to define underweight, normal weight, and overweight/obese, WHO growth standards [28]

### Commonly consumed prepared foods

Consumption of more than 100 dishes and other prepared food were reported by the adolescents, reflecting Gambian traditions [19, 23, 36, 37]. Typically, adolescents in both settings ate three meals each day: breakfast, lunch (afternoon meal) and dinner (evening meal). Table 2 highlights examples of less known prepared foods reflecting recent preferences in rural and urban areas, described using English and Wolof names. Mandinka names are provided in Supplementary file S2. The full detail of commonly prepared food, including those not associated with particular meals is in Supplementary file S3. Oil, particularly palm oil, and salt are usual key ingredients of savoury dishes, and sugar is often added to porridges, cream and drinks. White rice was the predominant staple food reported across all study settings, commonly consumed as the main meal at lunch, particularly in urban areas. In rural communities, adolescent girls frequently reported eating the same rice-based dish for breakfast, lunch, and dinner. Across both contexts, rice was typically accompanied by a variety of traditional stews and soups prepared with vegetables, fish, or groundnuts.

#### Breakfast (and dinner)

Breakfast foods in both settings were often also commonly eaten in larger potions for dinner. Breakfasts made from a range of cereals, served with sour cream or milk and often with sugar added, were common in both settings. Among urban adolescent girls this included proprietary breakfast cereals such as cornflakes and porridge made with oats, but also traditional dishes such as cassava porridge (*garri*) sweetened with sugar and served with or without nuts. In both urban and rural settings, sweetened couscous (c*hakry*) was commonly consumed. Regularly consumed in the rural settings were traditional rice porridges with or without added non-roasted groundnut (peanut) powder, or millet porridge (*rui* or *lahk)* (Table 2). Bread with sugar and water (sugar solution), and sour cream with or without sugar, consumed without accompaniment, were also simple breakfasts regularly eaten by adolescent girls in the rural setting.

In addition to the above cereal dishes, urban adolescent girls ate a wider range of breakfast choices served with traditional stews, bread, or rice. These included grilled meat, fried fish and sausage dishes, as well as more non-traditional foods such as mashed potato with egg, noodles, spaghetti and pasta, and pizza. Additionally, sandwiches made with fish, eggs or bean stew (s*auce nyebeh*, Table 2) were commonly consumed for breakfast in the urban areas but not in the rural settings.

#### Lunch

Traditional stews, made with meat or fish, starchy tubers, and other vegetables, most often served with rice, were commonly consumed for lunch in both settings. Leftover stews were often the accompaniment served with breakfast choices among the urban adolescents, mentioned above. There were stews mostly eaten in the urban setting. However, for other stews, meat and/or fish variations were eaten by the urban adolescent girls, and versions made with fish only were consumed by the rural adolescents.

Traditional stews predominantly consumed by the urban adolescents included different types of vegetable oil stew made with fish balls *(chu bullet)*, or fish (*chu jen*), and meat *(chu ganarr or chu yapa)*. Versions of stews (*chu)* with fish only, and of the national dish made with a tomato based sauce, vegetables, potatoes and/ or sweet potatoes, hot chilli, and with or without peanut butter) with meat or fish (*maffeh* [commonly known as *domoda* a Mandinka term but commonly used across tribes]) were stews eaten in both settings, depending on the main protein food added. Other stews featured okra (s*uper kanja*) or hibiscus leaf (*rangha bisap*) as well as other vegetables, meat, or fish. A lunchtime stew made with callaloo or sweet potato leaves, smoked fish, optional meat, onions, peppers, groundnut paste, and palm oil (*plasas*, Table 2), was predominantly reported by rural adolescents.

In addition to traditional stews, one pot rice dishes (*benachin)* made with meat or fish and vegetables (Table 2) were regularly consumed among the urban adolescent girls. Other one-pot rice dishes included *mbahal goor gigen* eaten in both settings (depending on whether meat or fish are added), and *mbahal guerteh*, distinguished by the addition of peanut powder, were more commonly eaten in the rural setting.

#### Drinks and snacks

Sugar-sweetened beverages were popular in both settings. These included traditional drinks with added sugar made from hibiscus leaves (*wonjo*) among the urban adolescents, and Baobab seeds (*juici bui*) in both settings. Proprietary, sweetened soft drinks were also consumed in the rural setting and sometimes added to the traditional drinks. Tea, coffee and *Ovaltine* (a proprietary malt drink made with milk) were also consumed. Sugar was added to tea, and evaporated milk was sometimes added. Rural adolescents also drank green tea (*ataya*) to which sugar was added.

A range of homemade cakes and pancakes were reported by urban adolescents, whereas shop-bought biscuits, sweets and chewing gum were consumed in both settings. A greater range of fruits were eaten by rural compared to urban adolescent girls (Table 2). Overall snacking between meals, other than consuming bread and butter and eating fruit, was much less common among the rural adolescents. By contrast, urban adolescents regularly snacked. Savoury snacks included a seafood one-pot stew with cassava and other vegetables (*ebbeh*, Table 2) and a seasoned minced fish dish, mostly bought from street vendors.

**Table 2:** Selected commonly consumed dishes, snacks and drinks, by urban and rural setting

Name^a^ (English name) Ingredients			
Popular in urban areas		Popular in rural areas	
Consumed for breakfast or in larger quantities for dinner (evening meal)			
*Sauci njabeh* (bean stew sandwich) Black eyed beans Cooking oil Onion Spring onion Garlic Black pepper Chilli pepper Seasoning cubes or sachets			*Rui* or *Lakh* (millet porridge); *Served with sour cream and milk* Millet Sugar Salt Optional: butter, nutmeg, grated fresh coconut
Consumed for lunch			
*Benachin* (one pot rice dish) Rice Meat (beef, mutton, or chicken) or fish (e.g. *kobo*, red snapper) Cooking oil Onion Spring onion Vegetables/ starchy staple of choice (e.g. casava, carrots, bitter tomato, aubergine, cabbage) Cherry tomatoes Optional tomato paste Chilli pepper Seasoning cubes or sachets			*Plasas* (green leaves stew); served with rice Green vegetable leaves of choice (e.g. callaloo, sweet potato or cassava leaves), Peanut powder or roasted peanuts Palm oil Fresh fish (lady fish) and smoked fish (e.g. *kobo*) Spring onions Locust beans Bitter tomatoes Scotch bonnet chilli pepper
Snacks and drinks			
*Ebbeh* (seafood one pot stew) Cassava Palm oil Smoked fish (usually *kobo*) Whole crab Shrimp Lime juice Tamarind juice Hot chillies Seasoning cubes			*Mbourou ak butter* (bread and butter)^b^
Range of fruits: Apple, orange, banana, jackfruit			Range of fruits: Ripe mango, raw Unripe mango, raw or boiled (salt, pepper, seasoning cube added) Pawpaw (papaya), cashew nuts *Kaba* (*Saba senegalensis*^3^) *Toll* (*Landolphia heudelotii*^3^)
*Tea ak mew* (tea with milk [optional] and sugar)			

^a^Local names are in Wolof language; Mandinka translations can be found in Supplementary file S2, ^b^Snacking between meals, other than consuming bread and butter and fruit, is uncommon in the rural areas; ^c^Botanical name (no commonly used English name)

#### Portion size, energy and nutrient composition

Reported portion size, and calculated energy and nutrient composition of the popularly consumed dishes, snacks and drinks consumed in each setting are in Supplementary file S4. The average reported portion size of *chu* was larger for rural compared to urban adolescent girls. This was reflected in the higher energy and carbohydrate content in a portion consumed in the rural setting. The higher fat content and vitamin A also reflected the greater quantity of vegetable oil and tomatoes used. However, the similar protein content indicates the proportionately smaller quantities of fish used in the rural setting. The high levels of fat in urban dishes, particularly one pot dishes such as *benachin* where vegetable oil is the main ingredient, were highly calorific with one portion providing over 1000 kcals. Traditional stews mostly eaten in the rural setting, such as *plasas* and *rangha bisap*, were lower in energy and fat, but were good sources of vitamin A. *Maffeh*, *plasas*, and millet *rui* were good sources of protein per portion.

As a common snack, portions of bread and butter were larger among the urban adolescent girls, and the large amounts of butter used contributed to high fat and vitamin A content. Snacks consumed in urban areas such as *ebbeh* and bean stew sandwich, in addition to main dishes, were good sources of protein. The high carbohydrate content in tea in both settings, and *wonjo* consumed by urban girls signals the large quantities of sugar added.

### Energy and nutrient intakes

#### Energy and nutrient intakes, by setting and nutritional status

Energy and nutrients intakes by rural and urban setting are shown in Table 3. Energy, protein, total fat, carbohydrate, iron, and vitamin C intakes were similar between settings. Average daily saturated fat and polyunsaturated fat intakes, however, were higher, and fibre intake lower, among urban compared to rural adolescent girls. Also lower among rural compared to urban adolescent girls were calcium (289.24 vs. 390.31 mg; *p* < 0.001), vitamin A (163.80 vs. 222.35 µg; *p* = 0.007), and vitamin D (1.33 vs. 1.86 µg ; *p* = 0.009) intakes.

**Table 3:** Comparison of energy and nutrient intake, by urban and rural setting, *N* = 203

		Median^a^ (Interquartile range)						
		All		Urban		Rural		*p*-value^b^
Energy, MJ^c^		9.14 (7.50, 11.16)		8.94 (7.35, 11.26)		9.18 (7.97, 11.03)		0.382
Protein, g		56.58 (43.34, 66.03)		55.84 (44.37, 68.23)		57.08 (43.00, 65.99)		0.929
Protein, % energy		10 (8, 12)		11 (9, 13)		10 (8, 12)		0.051
Total fat, g		105.37 (72.93, 134.72)		102.80 (69.55, 134.38)		106.91 (80.72, 136.45)		0.411
Total fat, % energy		43 (34, 51)		44 (34, 51)		41 (34, 51)		0.407
Saturated fat, g		15.02 (8.19, 24.25)		17.99 (14.03, 26.70)		7.79 (5.43, 15.15)		**< 0.001**
Saturated fat, % energy		6 (3, 10)		8 (6, 11)		3 (2, 7)		**< 0.001**
Polyunsaturated fat, g		4.47 (3.20, 6.50)		5.31 (3.86, 7.19)		3.34 (2.33, 4.99)		**< 0.001**
Polyunsaturated fat, % energy		2 (1, 3)		2 (2, 3)		1 (1, 2)		**< 0.001**
Carbohydrate, g		246.63 (210.86, 308.61)		237.87 (197.29, 289.29)		266.02 (232.25, 326.16)		0.467
Carbohydrate, % energy		46 (40, 55)		46 (39, 53)		47 (41, 55)		0.116
Total free sugars, g		23.15 (14.11, 35.57)		25.81 (16.26, 36.56)		21.42 (8.53, 34.62)		**0.036**
Total free sugars, % energy		4.01 (2.46, 6.69)		4.46 (2.84, 7.33)		3.47 (1.54, 5.92)		**0.008**
Dietary fibre, NSP^d^ (Englyst), g		16.45 (13.23, 20.40)		15.06 (12.26, 19.42)		17.67 (14.23, 22.29)		**< 0.001**
Calcium, mg		357.72 (280.20, 451.29)		390.31 (338.57, 493.18)		289.24 (224.81, 388.32)		**< 0.001**
Iron Fe, mg		8.95 (7.02, 11.41)		9.02 (7.02, 11.89)		8.71 (6.98, 10.99)		0.549
Vitamin A, retinol equivalents, µg^c^		203.62 (119.60, 334.18)		222.35 (145.30, 341.33)		163.80 (91.10, 304.56)		**0.007**
Vitamin D, µg		1.73 (0.80, 3.11)		1.86 (0.98, 3.74)		1.33 (0.22, 2.54)		**0.009**
Vitamin C, mg		33.73 (16.66, 66.22)		39.27 (18.87, 72.35)		28.01 (14.68, 61.40)		0.111

^a^Median and interquartile range, ^b^Rural-urban differences from Mann-Whitney U tests, bold type = statistically significant (*p* < 0.05), ^c^Megajoules, ^d^Non-starch polysaccharides, ^e^1 x retinol equivalent = 1 µg of retinol or 6 µg of β-carotene

The urban-rural comparisons, stratified by older (15–19 years) and younger (10–14 years) adolescents, are shown in Supplementary file S5. Findings show broadly the same pattern of urban-rural differences reported in Table 3, indicating that these differences are not driven by the younger age profile of the rural adolescent girls. However, they do also bring to light the suggestion that the urban-rural differences for energy, total fat intake, and total free sugars may be more important in the older than the younger adolescents (Supplementary file S5). Restricting the sample to the acceptable reporters also shows similar urban-rural patterns as for the whole sample (Supplementary file S5).

Guidelines for energy and nutrient intakes, drawing on international literature [38–44] in the absence of country specific values, and whether participants’ intakes met, were below or exceeded recommendations (according to the reference values chosen) are summarised in Table 4. It should be noted that the iron recommendation presented in Table 4 (30–60 mg/day) is the WHO therapeutic/supplementation guidance for populations with high prevalence of anaemia (whereby the prevalence of anaemia in infants and young children is ≥ 40% for three consecutive months in a year; as is the case for The Gambia) [15]. This is an alternative to standard dietary reference intake values, and should therefore be interpreted accordingly. Across settings, percentage energy intake from total fat and carbohydrates exceeded recommendations [39], and intakes of fibre were below recommended values [39]. While daily protein intakes exceeded WHO recommendations [38], recent advice acknowledges that protein intakes *≥* 15% of energy can be common among adolescents [40]. Despite higher intakes of vitamins A and D among urban adolescent girls compared with their rural counterparts, levels in both settings were below WHO recommended intakes for both vitamins [41].

**Table 4:** Dietary intake guidelines

Dietary component	Age group, years (where provided)	Recommended values per day^a^	Below, meets or exceeds the stated recommendations^b^	
			Urban	Rural
Energy, MJ^c^ [44]	Girls 10–18	7.1–8.4	Exceeds	Exceeds
Protein, g [38]	Adolescents	26–47	Exceeds	Exceeds
Protein, % energy	Adolescents	-	-	-
Total fat, g [44]	Girls 10–18	< 66–78	Exceeds	Exceeds
Total fat, % energy [39]	Adults	≤ 30^d^	Exceeds	Exceeds
Saturated fat, g [44]	Girls 10–18	< 21–24	Meets	Meets
Saturated fat, % energy [39]	Adults and children	< 10^d^ 10^e^	Meets	Meets
Polyunsaturated fat, g [44]	Girls 10–18	12–14	Below	Below
Polyunsaturated fat, % energy [39]	Population based estimate	6–10	Below	Below
Carbohydrates, g [44]	Girls 10–18	≥ 227–267	Meets	Meets
Carbohydrates, % energy [41]	Adults and children	45–60	Meets	Meets
Total free sugars, g [40]	Adults and children ≥ 10	≤ 50	Meets	Meets
Total free sugars, % energy [42]	Adults and children	< 5^d^ < 10^e^	Meets	Meets
Dietary fibre, NSP^f^ (Englyst), g [39]	≥ 10	≥ 25	Below	Below
Calcium, mg [44]	Girls 10–18	550–800	Below	Below
Iron Fe, mg [43]^g^	Children 5–12	30–60^g^	Below	Below
Vitamin A, retinol equivalents, µg^h^ [44]	Girls 10–18	500–600	Below	Below
Vitamin D, µg [44]	Girls 10–18	10	Below	Below
Vitamin C, mg [44]	Girls 10–18	30–40	Meets	Below
Fruits and vegetables, g [45]	≥ 10	≥ 400	Below	Below

^a^Recommended values are based on international energy and nutrient intake guidelines [38–44], used in the absence of Gambian-specific recommendations, ^b^according to the chosen references, ^c^Megajoules; ^d^WHO ‘Conditional’ recommendation [very low certainty evidence], ^e^WHO ‘Strong’ recommendation [moderate certainty evidence], ^f^Non-starch polysaccharides, ^g^The iron recommendation (30–60 mg/day) is the WHO therapeutic/supplementation guidance for populations with high prevalence of anaemia (whereby the prevalence of anaemia in infants and young children is ≥ 40% for three consecutive months in a year; as is the case for The Gambia) [15] rather than a standard dietary intake target, ^h^1 x retinol equivalent = 1 µg of retinol or 6 µg of β-carotene

Energy and nutrient intakes by nutritional status (stunting and weight status categories) are described in Supplementary file S6. Adolescent girls who experienced stunting reported lower saturated fat and calcium intakes compared to girls of normal height for age. Compared to those in normal, overweight, or obese weight status categories, underweight adolescent girls reported higher energy intakes (*p* = 0.015). Sensitivity analysis excluding participants classified as over reporters, showed the same pattern of higher median energy intakes among underweight adolescent girls (10.00 MJ [underweight], 8.86 MJ [normal weight], and 7.89 MJ [overweight or obese]; *p* = 0.009).

#### Energy and nutrient contribution of food groups

Supplementary file S7 shows the selected food groups and their contribution to energy and nutrient intakes. Meat and fish, particularly oily fish, provided 25% of protein intake. In line with being the country’s staple food, rice contributed almost 50% of dietary energy intake, due to providing 72% of carbohydrate intake, and strikingly 38% of protein intake, exceeding protein from meat and fish. Oils and spreading fats contributed nearly 20% of energy intake. Added sugar, sugary snacks, puddings, fruit, and dried fruit provided 60% of total sugar intake. In relation to the vitamins of interest, fish, fish products, and eggs were major contributors to dietary vitamin D. Although fruit and vegetables featured regularly in the food patterns described above, overall consumption was substantially less than the recommended 400 g or five portions per day [45]. However, fruit provided 53% and vegetables/ vegetable dishes 18% of vitamin C intake. Notably, locally prepared, traditional sugar-sweetened beverages, which included vitamin C-rich ingredients such as baobab, hibiscus, and tamarind, also contributed 18% of vitamin C intake. Vegetables, roots and tubers contributed 40% of vitamin A to the diet, and a further 15% came from meat and meat products. Bread, flours, and crackers provided 27% of calcium intake, and rice over 20%. Condiments, bread, flour and crackers, and vegetables/ vegetable dishes contributed 13–17% of iron intake.

## Discussion

To the authors’ knowledge, this study presents the first comprehensive report of food consumption and nutrient intake, including urban-rural comparisons and in relation to nutritional status, among adolescent girls in The Gambia. The findings reveal differences and similarities between the diets of adolescent girls in the two settings. Fried and processed foods featured highly in urban diets, whereas rural diets consisted mainly of cereal-based porridges and traditional stews. Overall, diets in both groups were energy-dense, but low in fruit and vegetables and consequently fibre, and deficient in iron. Excess fat and carbohydrate as a percentage of energy intake featured in the diets of both groups, and urban adolescent girls consumed significantly more saturated fat than rural peers. Despite greater intakes of micronutrients among the urban adolescents, vitamins A and D, and calcium deficiencies were evident for both urban and rural participants. Higher intakes of free sugars among urban adolescent girls, low vitamin C intakes among rural adolescent girls, and low calcium intakes among those with stunting were also notable disparities. White rice provided nearly half of total energy intake, and a significant amount of daily protein and calcium across settings. The predominance of sugar-sweetened beverages, and the importance of traditional sweetened drinks as a source of vitamin C, is a notable concern.

The observed diets among adolescent girls in The Gambia align with wider evidence for adolescent populations across SSA countries [7, 46]. Similar to findings from Senegal, Ghana and Nigeria [6, 47–49], and adding to previous frequency only data from The Gambia [17], the present study demonstrates that urbanisation is associated with higher consumption of fatty foods and sugar-sweetened beverages. Conversely, rural adolescents are more dependent on starchy staples and vegetable-based traditional stews, often lacking animal-source foods and fortified products that are more accessible in urban areas [50]. The predominance of rice as the staple source of energy and protein parallels dietary data from other African countries [5, 7, 23, 51]. A particularly notable finding was that rice accounted for 38% of total daily protein intake. Rice, however, is a lower-quality protein source due to its limited essential amino acid profile, particularly its low lysine content, compared with animal-source proteins [52, 53]. This reliance on protein from rice may reflect poor dietary diversity and limited access to nutrient-dense protein sources such as meat, fish, eggs, and dairy. Adolescence is a critical period, characterised by increased protein and micronutrient requirements to support rapid growth and development [54, 55]. Consequently, even where total protein intake appears adequate, poor protein quality may compromise these needs, and overall nutritional status in this population. The relatively high fat intake from oils and spreads is also consistent with transitions to increasing quantities of vegetable oil being used in traditional dishes [5, 7, 19, 49, 56]. Low fruit and vegetable consumption, below recommended intakes across both settings, is a persistent feature of adolescent diets in SSA [5, 7, 17, 51].

Low calcium and vitamin A intakes, exacerbated among rural participants, also reflects limited consumption of dairy products. Although fish, and to a lesser extent eggs, were an important source of vitamin D, consumption was not sufficient to meet recommended intakes. This pattern is consistent with evidence from rural Ghanaian adolescents where diets dominated by starchy staples and low consumption of dairy products, animal-source and fortified foods are associated with inadequate micronutrient intake [57, 58]. Exposure to sunlight, leading to synthesis in the skin is the best known source of vitamin D; however, despite year round sunshine, high prevalence of vitamin D insufficiency and deficiency have been reported in a number of African countries [59]. Lack of sunlight exposure and religious/ cultural clothing may be contributing factors [59], increasing the need to address the low dietary intakes of vitamin D and calcium reported here. The contribution of beverages to vitamin C intake was primarily driven by traditional drinks, rather than commercially fortified sugar-sweetened beverages. Traditional beverages prepared from vitamin C-rich local ingredients such as baobab pulp provide nutritional benefits [60]. However, nutrient retention may vary depending on preparation and storage [61], and addressing the large quantities of sugar added is an important consideration for health promotion messaging [42].

We could not find any comparative studies within the existing evidence base that have examined energy and nutrient intakes among adolescent girls living with stunting in SSA countries. In this study, higher energy intake reported by underweight adolescents compared to those in normal-weight and overweight/obese categories, is a pattern seen in other adolescent populations [62–64], though not universally observed [65, 66]. One possible explanation is that physical activity levels, elevated by daily activities such as walking long distances and household labour, particularly in rural contexts [16, 19], may increase energy demands. Sensitivity analyses excluding potential over reporters produced similar results, suggesting that the observed association is unlikely to be explained solely by reporting bias, although the potential for misreporting remains. This finding may also reflect mechanisms increasing energy demands beyond physical activity, contributing to undernutrition despite relatively higher reported energy intake. These include compensatory or catch-up growth following earlier nutritional deficits [70], and chronic infections, prevalent in many low-resource settings [71, 72]. In line with some SSA countries [50, 57, 62, 65], overweight and obesity prevalence among the adolescent girls in this study is relatively low. However, the high proportion of dietary energy derived from fats and refined carbohydrates, indicates a shift toward less healthy dietary patterns. In turn, these trends are reshaping traditional diets toward energy-rich, processed foods, while undernutrition remains prevalent [13, 14, 17, 56]. The findings emphasise the potential for the coexistence of chronic undernutrition and early, diet-related, noncommunicable disease risk seen in many West African countries undergoing rapid socio-economic change [56, 67].

### Strengths and limitations

This study has several important strengths. It provides a novel, comprehensive, comparative assessment of food consumption and nutrient intake among adolescent girls in rural and urban settings in The Gambia. The use of two interviewer-administered 24-hour multiple-pass recalls, adapted to the Gambian context and supported by extensive food vocabulary and portion-size aids, enhanced recall accuracy. The inclusion of both qualitative food descriptions and quantitative nutrient composition data provided a culturally grounded understanding of adolescents’ diets that reflects traditional and emerging dietary behaviours. The study also benefited from strong community participation and high response rates.

Certain limitations should be acknowledged. These cross-sectional findings preclude causal inference between dietary patterns and nutritional outcomes such as stunting or overweight due to the possibility of reverse causality, and potential residual confounding and measurement error. However, they provide topics for hypothesis testing in analytical studies. The assumption of independent observations underpinned the statistical analyses, and potential household- or village-level clustering were not explicitly taken into account. Shared dietary practices and food environments within households and communities may have influenced dietary intake patterns, potentially affecting variance estimates [68]. Sampling and multilevel analytical approaches that account for clustering are important considerations for future, large, analytical studies in The Gambia and similar settings [68, 69]. The 24-hour recall method, though widely used in dietary assessment, is subject to recall and social desirability biases [70], particularly in adolescents, who may forget or misreport items, such as snacks or foods consumed communally. Despite using two recalls, the study was not designed to fully capture day-to-day variation (and therefore habitual intake), or account for seasonal differences in food availability. Data collection during July and August, typically a lean season of depleted food supplies and income, may therefore represent a snapshot of dietary patterns during a period of heightened food insecurity [71, 72]. Validation against reference methods may be needed in this setting [70]. Formal inter-observer reliability testing was not conducted and is therefore acknowledged as a methodological limitation. The absence of visual portion size aids during the telephone recall might be considered a limitation. However, this was deemed to not compromise the depth or quantity of portion size information reported as the dietary information obtained from each recall was of comparable detail. In addition, participants were already familiar with the visual portion size aids (commonly used items such as drinking cups and ladles) used during the initial face-to-face recall, which likely enhanced consistency in reporting. Also, a limitation of this study is the use of nutrient composition data from non-Gambian food composition databases for some ingredients where local analytical data were unavailable. Bespoke recipes were created for all composite dishes; however, it is possible that selected proxy ingredients and cooking adjustment factors within the software may not exactly match the composition of local ingredients and local cooking methods. Consequently, a small degree of misclassification in estimated nutrient intake is possible, and the findings should be interpreted with this limitation in mind. Across the composition data sources, the common problem of incomplete micronutrient values [73], may have introduced uncertainty in estimates for the micronutrients reported in this study.

Our study focused on adolescent girls aged 10–19 years from two regions. By design, the study settings and participants were not intended to be statistically representative and therefore the findings may not generalisable. However, given the size and other contextual characteristics of the selected regions [24], the current study findings are potentially more indicative of the wider national picture than would be the case in larger, more geographically and food-diverse countries. Therefore, despite the limitations of representativeness, the study offers robust and contextually relevant evidence on the evolving nutritional challenges facing Gambian adolescent girls, providing a foundation for future policy, intervention development, and other study designs.

### Policy and research implications

The co-existence of high calorie but nutrient deficient diets in both settings, alongside the greater intake of saturated fat and sugar-rich foods in urban settings, reflects the potential for a triple burden of malnutrition risk typical in LMICs experiencing dietary and lifestyle shifts [56].

For policymakers, the study reinforces the urgency of developing a national adolescent nutrition strategy aligned with UNICEF and WHO recommendations [74, 75], and the integration of adolescent nutrition into national health information systems and surveillance frameworks. Our findings support recommendations for school-based micronutrient supplementation, school gardens, and other community-based food production schemes, and behaviour change interventions aimed at reducing consumption of sugar-sweetened beverages, other high-sugar and high-fat foods [3, 76]. Existing monitoring of dietary patterns, nutrient intake, and nutritional status indicators such as anaemia, BMI-for-age and stunting prevalences in The Gambia, which has focused on improving maternal, infant and young child nutrition [76], could ideally be extended to adolescent age-groups.

Future research could incorporate biochemical assessments. As a priority, 25-hydroxyvitamin D assessments would reflect total vitamin D stores from sunlight, diet, and supplements, and therefore accurately quantify the prevalence of deficiency [77]. Longitudinal cohort studies are needed to monitor how dietary behaviours evolve through adolescence and to identify critical windows for intervention. Emerging evidence suggests that better nutrition knowledge is associated with higher consumption of fruit, vegetables, fish, and eggs, whereas poorer nutrition literacy is linked to greater intake of high-calorie foods [87]. Our findings have already informed the development phase of a coproduced intervention with youth at the centre, integrating nutrition-specific (e.g. interactive nutrition education; food supplementation) and nutrition-sensitive components (e.g. school gardening) that could enhance dietary diversity in participating areas [78]. Care is needed so that actions addressing unfavourable diets mitigate against the importance of rice for protein and iron, and traditional sweetened drinks for vitamin C, for example, to avoid exacerbating inequalities. Ultimately, improving adolescent nutrition in The Gambia represents both a health and development imperative, contributing to progress toward the Sustainable Development Goals (SDGs 2, 3, and 5) [79].

## Conclusion

Adolescence is a pivotal period for catch-up growth and cognitive development, and investing in adolescent nutrition will not only improve immediate wellbeing but also yield long term benefits for subsequent generations. This study describes similarities and differences in rural and urban diets, and in relation to nutritional status, among adolescent girls in The Gambia. Popularly consumed foods and typical portion sizes were described and, overall, diets were high-calorie, low in fruit and vegetables and lacking adequate fibre. Inadequate micro-nutrient intakes (vitamins A and D, calcium and iron) were reported in both settings. Additional setting-specific concerns included higher processed foods, sugar and saturated fat intakes among the urban participants, and low vitamin C among rural respondents. Adolescent girls living with stunting had lower calcium intakes than peers who were not. The findings are context-specific and descriptive; however, they resonate with other West African contexts, and support the need for targeted, intervention strategies to address malnutrition among adolescent girls and the potential trend towards a triple burden of malnutrition risk. Such actions in The Gambia need to be sensitive to urban-rural nuances and driven by concerted policy focus.

## Supplementary Information

Supplementary Material 1: Supplementary File S1: STROBE-nut checklist for cross-sectional studies.

Supplementary Material 2: Supplementary File S2: English - Mandinka - Wolof vocabulary: selected dishes, beverages and snacks.

Supplementary Material 3: Supplementary File S3: Commonly consumed dishes and other prepared food.

Supplementary Material 4: Supplementary File S4: Portion size, energy and nutrient composition of the most commonly consumed foods, by setting.

Supplementary Material 5: Supplementary File S5: Comparison of energy and nutrient intake by urban and rural - setting sensitivity analyses.

Supplementary Material 6: Supplementary File S6: Comparison of energy and nutrient intake by nutritional status (weight status and stunting categories).

Supplementary Material 7: Supplementary File S7: Food consumption in selected food groups and percentage contribution to energy and macronutrient intake.

## Data Availability

All relevant data and analysis supporting the findings of this study are provided in this article and the accompanying supplementary files.

## Abbreviations

EIrep:BMRest: Ratio of reported energy intake (EIrep) to estimated basal metabolic rate (BMRest)
LGAs: Local Government Areas
MRCG: Medical Research Council Unit The Gambia at the London School of Hygiene & Tropical Medicine
SSA: Sub-Saharan Africa/African
STROBE-NUT: Strengthening the Reporting of Observational Studies in Epidemiology- Nutritional Epidemiology
WHO: World Health Organisation
UNICEF: United Nations Children’s Fund
