# Supplementary material for: Food consumption and nutrient intake among adolescent girls in The Gambia: a cross-sectional study with urban-rural and nutritional status comparisons
Source: BMC Nutr. 2026 Jul 17;12:215. doi: 10.1186/s40795-026-01430-0 (PMC13656378; doi:10.1186/s40795-026-01430-0)
Supplement: Supplementary file 2 [file 40795_2026_1430_MOESM2_ESM.docx]

**Supplementary file 2.**

**English - Mandinka - Wolof vocabulary: selected dishes, beverages and snacks**

| English | Mandinka | Wolof |
| --- | --- | --- |
| Apple | Pomo | Pum |
| Banana | Banano | Banana |
| Baobab fruit juice | Sitajio | Ndohi bui |
| Baobab with steamed rice-sweetened | Nan buru | Nan buru |
| Black-eyed beans stew | Sauce Suso | Sauce Nyebeh |
| Bread and butter | Mburo butter | Mburu ak butter |
| Bread with sugar and water | Ponsewo | Ponseh |
| Couscous or millet steamed-sweetened | Chakry | Ckakry |
| Egg sandwich | Mburo niŋ-sisakilo | Mburu ak nen |
| Fish and lime stew | Caldu | Caldu |
| Fry fish with gravy | Nyewo fryriŋo | Feeril |
| Green leaves sauce | Jambo duraŋo | Plasas-hobi patas |
| Green tea | Ataya | Ataya |
| Grilled chicken | Sisay Janiriŋo | Afra ganarr |
| Grilled meat | Soubu Janiriŋo | Afra yapa |
| Groundnut, peanut fresh | Tio | Guerteh |
| Groundnut, peanut roasted | Tia yirariŋo | Guerteh chaff |
| Homemade fried chips | Chips | Chips |
| Mango | Duto | Mango |
| Mashed potato with eggs | Mashed potato | Mashed potato |
| Okra stew | Soupa Kanja | Soupa kanja |
| Orange | Oranso | Orange |
| Palm oil stew | Suss teŋ tulo | Chuwi dewtirr |
| Peanut paste stew | Tia duraŋo | Maffeh guerteh |
| Plain omelette | Omeletto | Omelette |
| Plain pancakes made with flour or millet | Pankato | Pancake |
| Plain rice thick porridge | Mani mono | Ruiyi malo |
| Potato sandwich | Mburo niŋ Pompitaro | Mburu ak pompitar |
| Rice, thick porridge with groundnuts | Tiakere churo | Churrah guerteh |
| Rice-based dish | Nyewo Nyangkataŋo | Mbahal goor gigen’ |
| Rice-based dish with peanut powder | Nyankantaŋo | Mbahal guerteh Nyangkatang |
| Rice-based dish with vegetable oil sauce | Benechino | Benachin |
| Sardine sandwich | Mburo niŋ sardino | Mburu ak sardine |
| Sausage stew | Sausage sauce so | Sauce sausage |
| Seafood dish | Ebbeh | Ebbeh |
| Sorrel ice/drink | Wonjo | Wonjo |
| Sour cream | Nono | Sow |
| Sour leaf or sorrel stew | Kucha duraŋo | Maffeh bissap |
| Steamed fish sandwich | Steamed fish | Steamed fish |
| Steamed millet powder | Futo | Chereh |
| Thin gruel millet porridge | Sanyo mono | Ruiyi duguub |
| Vegetable oil stew | Suss tulo | Chuwi dewlin |
| Wheat flour stew | Duraŋ farriŋo | Sauce farring |
