# Supplementary material for: Food consumption and nutrient intake among adolescent girls in The Gambia: a cross-sectional study with urban-rural and nutritional status comparisons
Source: BMC Nutr. 2026 Jul 17;12:215. doi: 10.1186/s40795-026-01430-0 (PMC13656378; doi:10.1186/s40795-026-01430-0)
Supplement: Supplementary file 3 [file 40795_2026_1430_MOESM3_ESM.docx]

**Supplementary file 3.**

**Commonly consumed dishes and other prepared food**

| Commonly consumed for breakfast and in larger portions for dinner | | |
| --- | --- | --- |
| **Traditional name (English translation if needed)** | **Main ingredients** | **Setting where most popular (urban, rural or both)** |
| **Served with sour cream or milk** |  |  |
| *Cornflakes* | Cornflakes, milk, with/without sugar | Urban |
| *Garri* (cassava porridge*)* | Cassava flour, milk, sugar, with or without nuts | Urban |
| *Nan buru* (baobab with rice-sweetened) | Baobab seeds, rice, milk powder, condensed milk, milk liquid, coco nut, essence, sugar, nut meg, with or without fruits | Urban |
| *Oat porridge* | Rolled oats, milk, sugar | Urban |
| *Chakry* (sweetened couscous) | Couscous, milk, sour cream, sugar, milk, sour cream, essence, | Urban and rural |
| *Churah* *guerteh* (rice and groundnut porridge) | Rice, peanut powder, sugar, salt | Rural |
| *Churah* *bou* *weeh* (rice porridge) | Rice, sugar, salt | Rural |
| *Rui* or *Lakh* (millet porridge) | Millet powder, sugar, salt, with/without (butter, nutmeg, coconut) | Rural |
| *Chereh* (steamed millet powder) -plain or sweetened | Millet powder, salt or sugar, water | Rural |
| *Ponseh* (bread with sugar and water) | Bread, sugar, with/without sour cream/milk | Rural |
| *Sow* (sour cream with/without sugar and milk) | Sour cream, with/without sugar/milk  Served on its own | Rural |
| **Served with stews, bread or rice** |  |  |
| *Afra* (grilled meat) | Beef, mutton, or chicken, onions, seasonings | Urban |
| *Chawarma* (wrap) | Wheat flour, choice of meat (beef, mutton or chicken), seasonings | Urban |
| *Chips* (homemade fried chips) | Potatoes, cooking oil, salt | Urban |
| *Feeril* (fried fish with gravy) | Fish, cooking oil, onions, black pepper, garlic, spices, seasonings  Also served with chips or spaghetti | Urban |
| *Mashed potato* with egg | Potatoes, boiled eggs, mayonnaise, with/ without mixed vegetables, seasonings | Urban |
| *Noodles* - plain or with meat or fish | Noodles, choice of fried meat or fish or boiled eggs and/ or sausage, onions, black pepper seasonings | Urban |
| *Pizza* | Wheat flour, choice of beef, mutton, chicken, mixed vegetables, cheese  No accompaniment | Urban |
| *Sauce* *ganarr* *ak macroni* (chicken and pasta stew) | Pasta, chicken, onions, cooking oil, black pepper, garlic, seasonings | Urban |
| *Sauce* *sausage* (sausage stew) | Sausage, onions, cooking oil, seasonings, spices, with or without mixed vegetables  Also served with chips or spaghetti | Urban |
| *Spaghetti* - plain or with sardines | Spaghetti, sardines, seasonings | Urban |
| **Sandwiches** |  |  |
| Steamed fish sandwich | Minced fish, onions, cooking oil, seasonings, spices | Urban |
| Sardine sandwich | Sardine, with/without choice of boiled egg, canned beef, boiled potato, or avocado, mayonnaise, onions, black pepper, seasonings | Urban |
| Egg sandwich | Boiled egg, bread, mayonnaise or butter, black pepper, seasoning cube | Urban |
| Potato sandwich | Boiled potato, choice of spread (butter or mayonnaise), with/without boiled egg, black pepper, seasoning cube | Urban |
| Spread sandwich | Bread, choice of spread (butter, mayonnaise, chocolate) | Urban |
| Plain omelette sandwich | Eggs, cooking oil, onions, black pepper, seasonings | Urban |
| Beans stew sandwich | Beans, cooking oil, onions, seasonings | Urban |
| **Commonly consumed for lunch** | | |
| **Traditional name (English translation)** | **Main ingredients** | **Setting where most popular (urban, rural or both)** |
| **Stews served with rice** |  |  |
| *Caldu* (fish and lime stew) | Fish of choice, lime, onions, black pepper, with/without vegetables, spices, seasonings | Urban |
| *Chu bullet* (vegetable oil stew with minced fish balls) | Minced fish made in balls, cooking oil, onions, spring onions, tomato cherry, with/without tomato paste, vegetables of choice, seasoned with black pepper, garlic, seasoning cubes or sachets, chilli pepper | Urban |
| *Chu kong* (vegetable oil stew with catfish) | Smoked catfish, cooking oil, onions, spring onions, tomato cherry, tomato paste, vegetables of choice, black pepper, garlic, seasonings | Urban |
| *Sauce bullet* *yapa* *ak* *macroni* (meatball and pasta stew) | Pasta, minced meat (beef or mutton) made into balls, onions, spring onions, vegetables of choice, seasonings, spices | Urban |
| *Chu* or *Yassa* (vegetable oil stew) | Fish, meat (beef, mutton [*Chu yapa*], or chicken [*Chu* *ganarr*/ *Yassa ganarr]*), cooking oil, onions, with/without tomato paste/tomato cherry, vegetables of choice, garlic, black pepper, seasonings, chilli pepper | Urban when cooked with meat and\or fish; rural when cooked with fish |
| *Chu* *dewtir* (palm oil stew) | Palm oil meat (beef, mutton, or chicken) or fish, tomato cherry, tomato paste, onions, spring onions, vegetables of choice, black pepper, garlic, chilli pepper, seasonings | Urban when cooked with meat; rural when cooked with fish |
| *Sauce farring* (wheat flour stew) | Wheat flour, fish, meat (beef, mutton, or chicken) cooking oil, onions, tomato puree, vegetables of choice, seasonings | Urban when cooked with meat; rural when cooked with fish |
| *Maffeh* *gurrteh* (peanut paste stew) | Roasted peanut butter, fresh/salted fish *[Maffeh guerteh* bou *jen]* or meat (beef, mutton [*Maffeh guerteh bou yapa*], or chicken [*Maffeh guerteh bou ganarr*]), tomato cherry, tomato paste, onions, with/ without locust beans, seasonings, vegetables of choice | Urban when cooked with meat; rural when cooked with fish |
| *Rangha* *bissap* (hibiscus leaves stew) | Hibiscus leaves, fish of choice, locust beans, okra, tomatoes, spices, lime | Urban when cooked with meat; rural when cooked with fish |
| *Super kanja* (okra stew with fish) | Okra, with/without molokhia/jute leaves, choice of beef, mutton, chicken, and/ or fish, palm oil, locust beans, fresh and smoked fish, chilli pepper, seafoods of choice | Urban when cooked with meat; rural when cooked with fish |
| *Plasas* (green leaves stew) | Green vegetable leaves of choice (e.g. callaloo), sweet potato and cassava leaves, peanut powder or roasted peanut, palm oil, fresh and smoked fish, choice of meat (beef, mutton, or chicken; optional), spring onions, locust beans, bitter tomatoes, Scotch bonnet chilli pepper | Rural |
| **One pot rice dish** |  |  |
| *Benachin* (one pot rice dish) | Rice, choice of beef, mutton *[Benachin yapa*],, chicken *Benachin ganarr]*, or fish *[Benachin jen]*, cooking oil, onions, spring onions, vegetables of choice, tomato cherry, with/without tomato paste, chilli pepper, seasonings | Urban |
| *Mbahal goor gigen* (one pot rice dish) | Rice, choice of beef, mutton chicken, or fish*,* cooking oil, onions, spring onions, minced fish made into balls, tomato cherry, tomato paste, vegetables of choice, locust beans, tamarind, black pepper, garlic, chilli pepper, seasonings | Urban when cooked with meat; rural when cooked with fish |
| *Mbahal guerteh* (one pot rice dish with peanut powder) | Rice, peanut powder, locust beans, spring onions, smoked and salted dry fish, okra whole, bitter tomatoes, Scotch bonnet chilli pepper | Rural |
| **Dishes not consumed at a specific meal** | | |
| **Traditional name (English translation)** | **Main ingredients** | **Setting where most popular (urban, rural or both)** |
| *Sauce yapa*/*ganarr* (vegetable oil stew) | Beef or mutton [*Sauce yapa*] and/ or chicken [*Sauce ganarr*], onions, cooking oil, vinegar, mustard, mixed vegetables of choice, black pepper, garlic, seasonings, chilli peppers  Served with vermicelli, couscous, rice, or bread | Urban |
| *Sauce* *yapa* *ak macroni* (meat and pasta stew) | Beef or mutton, pasta, onions, cooking oil, with/ without tomato paste, black pepper, garlic, seasonings  Served with rice or bread (optional) | Urban |
| *Pepper soup* | Choice of meat (beef, mutton [*Pepper soup bi yapa*], chicken [*Pepper soup bi ganarr*]) or fish (*Pepper soup bi jen*), onions, potatoes, with/without tomato paste, vegetables of choice, spices, seasonings  Served with rice, bread, or millet | Urban |
| **Commonly consumed with meals or as a snack** | | |
| **Traditional name (English translation)** | **Main ingredients** | **Setting where most popular (urban, rural or both)** |
| **Drinks** |  |  |
| *Wonjo* (hibiscus leaves drink) | Hibiscus leaves, sugar, with/without mint and/or Vimto drink | Urban |
| *Juice and drinks* | Juice of choice (fresh juice, sachet juice), soft drinks, coconut water, milk, Ovaltine, tea, coffee | Urban and rural [soft drinks urban only] |
| *Juici* *bui* (Baobab juice) | Baobab fruit, milk, sugar | Urban and rural |
| *Tea ak mew* (Tea with milk) | Black tea leaves with milk (optional) and sugar | Urban and rural |
| *Ataya* (green tea) | Green tea leaves, sugar, with or without mint | Rural |
| **Sweet snacks** |  |  |
| *Cakes* (groundnut cake, rice cake, pancake) | Plain/pancake (flour, sugar, eggs, butter, milk, baking powder, essence flavour, salt, cooking oil and millet for pancake), groundnut cake (peanuts, milk, sugar), rice cake (rice, sugar, milk, butter) | Urban |
| *Biscuits* | *Bawdoko*, *baba* [plain slightly sweetened biscuits], short bread biscuits | Urban and rural |
| *Sweets* | Mints, candies, chocolate/ sweet bars, chewing gum | Urban and rural |
| **Savoury snacks** |  |  |
| *Ebbeh* (seafood one pot stew) | Cassava, palm oil, smoked fish, crabs, shrimps, lime, tamarind, hot chillies, seasoning cubes | Urban |
| *Fish pie* | Flour, minced fish, mixed vegetables, seasonings, spices | Urban |
| *Mbourou* *ak* *butter* (bread and butter) |  | Urban and rural |
| *Guerteh* (peanuts) | Plain or roasted | Rural |
| **Fruit** |  |  |
| Apple orange, banana, jackfruit | Eaten raw | Urban |
| Ripe mango (raw), unripe mango (raw or boiled), pawpaw (papaya), cashew nuts, *Kaba (Saba senegalensis^1^)*  *Toll (Landolphia heudelotii^1^)* | Salt, pepper, seasoning added to boiled mango | Rural |

^1^Botanical name (no commonly used English name)
