# Supplementary material for: Food consumption and nutrient intake among adolescent girls in The Gambia: a cross-sectional study with urban-rural and nutritional status comparisons
Source: BMC Nutr. 2026 Jul 17;12:215. doi: 10.1186/s40795-026-01430-0 (PMC13656378; doi:10.1186/s40795-026-01430-0)
Supplement: Supplementary file 4 [file 40795_2026_1430_MOESM4_ESM.docx]

**Supplementary file 4.**

**(i) Portion size, energy and nutrient composition of the most commonly consumed foods, by setting**

|  | N (%) consumers | Portion size (g) | Energy, Kcal^1^ | Protein, g | Carbohydrate, g | Fat, g | Vitamin A, retinol equivalents, µg^2^ |
| --- | --- | --- | --- | --- | --- | --- | --- |
|  |  | ***Median (Inter quartile range)*** | | | | | |
| Prepared dishes, snacks and drinks | | | | | | | |
| Commonly consumed among urban and rural participants | | | | | | | |
| *Chu or Yassa* (vegetable oil stew with fish)  *Urban*  *Rural* | 40  (19)  41  (20) | 263  (176; 371)  400  (225; 573) | 514  (346; 694)  758  (390; 1065) | 5  (3; 6)  7  (3; 10) | 11  (7; 15)  16  (8; 22) | 50  (34; 68)  74  (38; 104) | 25  (13; 42)  44  (24; 63) |
| Bread and butter  *Urban*  *Rural* | 75  (36)  20  (10) | 90  (90; 127)  70  (70; 85) | 303  (303; 377)  210  (210; 214) | 7  (7; 9)  6  (6; 6) | 38  (38; 46)  31  (31; 31) | 14  (14; 14)  7  (7; 7) | 157  (157; 157)  39  (39; 39) |
| Tea with milk and sugar, mls  *Urban*  *Rural* | 71  (34)  21  (11) | 270  (270; 273)  260  (228; 265) | 59  (57; 71)  71  (54; 71) | 1  (1; 1)  1  (1; 2) | 11  (11; 14)  14  (11; 14) | 1  (1; 1)  1  (1; 1) | 16  (16; 16)  16  (14; 16) |
| Predominantly consumed among urban participants | | | | | | | |
| *Chu/ Yassa ganarr* (vegetable oil stew with chicken) | 32  (15) | 294  (147; 432) | 741  (371; 890) | 12  (6; 20) | 14  (7; 19) | 71  (35; 82) | 15  (7; 27) |
| *Benachin jen*  (one pot rice dish with fish) | 23  (11) | 281  (180; 480) | 1303  (882; 2089) | 19  (12; 30) | 69  (44; 111) | 116  (73; 185) | 1  (0; 1) |
| *Benachin yapa* (one pot rice dish with meat) | 15  (7) | 398  (349; 600) | 1361  (1193; 2052) | 39  (34; 59) | 65  (57; 98) | 105  (92; 158) | 22  (20; 40) |
| *Ebbeh* (seafood one pot stew) | 53  (25) | 152  (76; 152) | 239  (120; 239) | 8  (4; 8) | 40  (20; 40) | 6  (3; 6) | 8  (4; 8) |
| Beans stew sandwich | 41  (20) | 131  (65; 140) | 284  (284; 284) | 12  (12; 12) | 50  (20; 50) | 4  (1; 4) | 27  (17; 27) |
| *Wonjo* (hibiscus leaves drink), mls | 36  (17) | 126  (126; 126) | 134  (134; 134) | 0 | 34  (34; 34) | 0 | 0 |
| Predominantly consumed among rural participants | | | | | | | |
| *Maffeh guerteh bou j*en (peanut paste stew with fresh and salted fish) | 58  (28) | 275  (183; 366) | 621  (511; 1020) | 28  (23; 46) | 14  (11; 22) | 50  (42; 80) | 34  (27; 54) |
| *Plasas* (Green leaves stew with callaloo leaves, peanut powder and fish) | 18  (9) | 283  (150; 463) | 326  (173; 455) | 22  (12; 31) | 7  (4; 8) | 24  (13; 33) | 333  (177; 465) |
| *Rangha bissap* (hibiscus leaves stew) | 15  (7) | 79  (59; 138) | 134  (67; 235) | 12  (6; 22) | 4  (2; 7) | 8  (4; 14) | 161  (80; 263) |
| *Rui* or *Lakh* (millet porridge) | 26  (13) | 574  (397; 574) | 14  (10.; 14) | 117  (81; 117) | 6  (6; 6) | 3  (3; 3) | 2  (2; 2) |

^1^Energy expressed in kilocalories ; ^2^1 retinol equivalent = 1 μg of retinol or 6 μg of β-carotene.

**(ii) Example recipes**

| Name (English name)  Recipes (with typical ingredient amounts to serve 4) | | |
| --- | --- | --- |
| **Commonly consumed in urban areas** | **Commonly consumed in rural areas** | **Commonly consumed in rural and urban areas** |
| *Yassa ganarr* (vegetable oil stew with chicken)  *900g chicken^1^*  2 tbsp cooking oil  4 medium onions,  *2 tsp mustard^1^*  *150g mixed vegetables (frozen^1^*  *1 medium whole garlic^1^*  *1 seasoning cube^1^*  *2 whole Scotch bonnet^1^*  *1 tsp black pepper^1^*  *2 medium lemons^1^*  *1 tbsp vinegar^1^*  500mls water  Method:  1. Fry the chicken in the oil until brown  2. Remove the chicken. Season and fry the chop onions until brown  3. Add water and simmer  4. Add chicken and mixed vegetables and leave to cook  5. Serve with rice | *Maffeh guerteh bou jen* (peanut paste stew with fresh and salted fish)  150g fresh *kobo* fish  *50g salted cod^1^*  *2tbsp roasted peanut butter^1^*  *75g cherry tomatoes^1^*  *2 tsp tomato paste^1^*  1 small onion  1 tbsp locust beans  *1 seasoning cube^1^*  1 medium aubergine  *2 medium bitter tomatoes^1^*  *2 medium potatoes^1^*  *2 whole Scotch bonnet^1^*  1l water  Method:  1. Add water to cooking pot, then add fresh fish and peanut butter  2. Leave to boil, remove fish when cooked  3. Chop onions and pound with locust beans, tomatoes and seasonings  3. De-bone the fish and add to the cooking pot with the other ingredients  4. Simmer until cooked  5. Serve with rice | *Chu jen* (vegetable oil stew with fish)  200g fresh fish  2tbsp cooking oil  4 medium onions,  *2tsp tomato paste^1^*  *75g cherry tomatoes^1^*  *2 medium carrots^1^*  1 medium aubergine  1 medium cassava  *1 medium whole garlic^1^*  *1 tsp black pepper^1^*  *1 seasoning cube^1^*  *1 whole Scotch bonnet^1^*  500mls water  Method:  1. Fry the fish in the oil until brown  2. Remove the fish. Chop onions and pound with tomatoes and seasonings then fry until brown  3. Add water and simmer  4. Add all vegetables and fish and leave to cook  5. Serve with rice |

^1^Ingredients in the italics are not in the food composition table of The Gambia (which mainly focuses on prepared dishes), but are assumed to have the same composition as their Gambian counterpart
