# Supplementary material for: Food consumption and nutrient intake among adolescent girls in The Gambia: a cross-sectional study with urban-rural and nutritional status comparisons
Source: BMC Nutr. 2026 Jul 17;12:215. doi: 10.1186/s40795-026-01430-0 (PMC13656378; doi:10.1186/s40795-026-01430-0)
Supplement: Supplementary file 5 [file 40795_2026_1430_MOESM5_ESM.docx]

**Supplementary file 5. Comparison of energy and nutrient intake, by urban and rural setting – sensitivity analyses**

**(i) Comparison of energy and nutrient intake, by urban and rural setting, stratified by age group (older adolescents vs younger adolescents), N=203**

|  | Median^1^  (Interquartile range) | | | | | |
| --- | --- | --- | --- | --- | --- | --- |
| Energy and nutrient | **Older adolescents**  (15 to 19 years) | | | **Younger adolescents**  (10 to14 years) | | |
|  | **Urban** | **Rural** | **p-value^2^** | **Urban** | **Rural** | **p-value^2^** |
| Energy, MJ^3^ | 8.86  (7.63, 10.41) | 10.05  (9.00, 11.14) | **0.015** | 9.15  (7.27, 11.40) | 9.54  (8.24, 11.05) | 0.338 |
| Protein, g | 51.49  (41.12, 69.15) | 54.39  (42.47, 65.78) | 0.621 | 53.36  (44.85, 60.99) | 55.94  (41.76, 65.85) | 0.982 |
| Protein, % energy | 11.00  (9.00, 13.00) | 10.00  (7.00, 12.00) | 0.104 | 10.00  (8.00, 13.00) | 10.00  (8.00, 12.00) | 0.453 |
| Total fat, g | 97.48  (66.32, 123.98) | 119.04  (95.59, 151.36) | **0.018** | 119.55  (75.11, 154.14) | 97.50  (73.09, 121.76) | 0.142 |
| Total fat, % energy | 42.00  (33.00, 50.00) | 46.00  (38.00, 54.00) | 0.081 | 47.00  (40.00, 56.00) | 38.00  (32.00, 47.00) | **0.006** |
| Saturated fat, g | 18.71  (13.37, 26.97) | 6.49  (4.92, 11.18) | **<0.001** | 17.55  (14.61, 25.89) | 7.85  (5.88, 17.52) | **<0.001** |
| Saturated fat, % energy | 8.00  (6.00, 12.00) | 3.00  (2.00, 5.00) | **<0.001** | 8.00  (6.00, 10.00) | 3.00  (2.00, 9.00) | **<0.001** |
| Polyunsaturated fat, g | 5.31  (3.86, 7.16) | 3.13  (2.10, 4.65) | **<0.001** | 5.32  (3.81, 7.99) | 3.26  (2.35, 4.86) | **<0.001** |
| Polyunsaturated fat, % energy | 2.00  (2.00, 3.00) | 1.00  (1.00, 2.00) | **<0.001** | 3.00  (2.00, 3.00) | 1.00  (1.00, 2.00) | **<0.001** |
| Carbohydrate, g | 240.20  (200.04, 281.85) | 240.93  (227.66, 304.22) | 0.271 | 214.86  (192.19, 291.77) | 273.13  (233.84, 336.41) | **<0.001** |
| Carbohydrate, % energy | 47.00  (41,00, 56.00) | 45.00  (39.00, 51.00) | 0.203 | 42.00  (37.00, 49.00) | 51.00  (43.00, 56.00) | **<0.001** |
| Total free sugars, g | 23.15  (16.32, 33.02) | 14.82  (5.58, 22.04) | **<0.001** | 28.18  (15.46, 39.23) | 25.48  (15.08, 42.82) | 0.717 |
| Dietary fibre, NSP (Englyst), g^4^ | 15.14  (12.32, 19.56) | 17.20  (14.81, 19.99) | **0.049** | 14.85  (12.65, 18.99) | 19.39  (13.52, 24.97) | **0.008** |
| Calcium, mg | 393.03  (338.70, 473.24) | 260.43  (201.66, 356.68) | **<0.001** | 376.69  (327.16, 510.47) | 295.82  (225.60, 346.28) | **<0.001** |
| Iron Fe, mg | 9.22  (7.91, 11.61) | 9.15  (7.49, 11.40) | 0.762 | 8.20  (6.07, 12.51) | 8.26  (6.82, 11.06) | 0.853 |
| Vitamin A, retinol equivalents, µg^5^ | 239.36  (162.99, 337.60) | 201.21  (102.30, 341.29) | 0.285 | 230.99  (132.02, 423.96) | 163.05  (97.52, 262.73) | 0.075 |
| Vitamin D, µg | 1.93  (0.95, 3.57) | 1.80  (0.82, 2.52) | 0.182 | 1.85  (1.20, 3.93) | 1.01  (0.04, 2.92) | **0.028** |
| Vitamin C, mg | 40.25  (21.04, 66.32) | 36.46  (20.09, 61.03) | 0.443 | 38.97  (14.52, 74.07) | 22.58  (14.05, 62.02) | 0.547 |

^1^Median and interquartile range; ^2^Rural-urban differences from Mann-Whitney U tests, bold type = statistically significant (p<0.05); ^3^Megajoules; ^4^Non-starch polysaccharides; ^5^1 retinol equivalent = 1 μg of retinol or 6 μg of β-carotene**.**

**(ii) Comparison of energy and nutrient intake, by urban and rural setting, acceptable reporters, N=169**

|  | Median^1^  (Interquartile range) | | | |
| --- | --- | --- | --- | --- |
|  | **All** | **Urban** | **Rural** | **p-value^2^** |
| Energy, MJ^3^ | 9.14  (7.74, 10.62) | 8.98  (7.57, 10.32) | 9.54  (8.54, 10.84) | **0.020** |
| Protein, g | 53.90  (44.31, 65.10) | 53.09  (45.31, 64.85) | 55.43  (42.80, 65.48) | 0.849 |
| Protein, % energy | 10.00  (8.00, 12.00) | 11.00  (9.00, 13.00) | 10.00  (8.00, 12.00) | 0.069 |
| Total fat, g | 105.47  (72.95, 129.64)) | 105.40  (71.09, 129.79) | 105.54  (74.14, 131.54) | 0.856 |
| Total fat, % energy | 44.00  (34.00, 51.00) | 44.00  (36.00, 51.00) | 40.00  (32.00, 50.00) | 0.194 |
| Saturated fat, g | 14.98  (7.66, 22.10) | 18.87  (14.46, 26.51) | 6.94  (5.44, 11.89) | **<0.001** |
| Saturated fat, % energy | 6.00  (3.00, 10.00) | 8.00  (6.00, 11.00) | 3.00  (2.00, 5.00) | **<0.001** |
| Polyunsaturated fat, g | 4.73  (3.06, 6.52) | 5.68  (4.35, 7.29) | 3.23  (2.21, 4.48) | **<0.001** |
| Polyunsaturated fat, % energy | 2.00  (1.00, 3.00) | 2.00  (2.00, 3.00) | 1.00  (1.00, 2.00) | **<0.001** |
| Carbohydrate, g | 242.51  (208.41, 303.73) | 230.27  (196.52, 281.19) | 263.70  (228.22, 321.84) | **<0.001** |
| Carbohydrate, % energy | 46.00  (40.00, 55.00) | 45.00  (39.00, 53.00) | 49.00  (41.00, 57.00) | **0.043** |
| Total free sugars | 23.20  (14.95, 35.12) | 25.45  (16.46, 35.19) | 21.53  (41.23, 56.83) | 0.155 |
| Total free sugars, % energy | 4.00  (3.00, 7.00) | 5.00  (3.00, 7.00) | 4.00  (2.00, 6.00) | 0.061 |
| Dietary fibre, NSP (Englyst), g^4^ | 16.28  (13.23, 20.51) | 14.42  (12.39, 19.01) | 17.63  (14.14, 22.42) | **<0.001** |
| Calcium, mg | 352.17  (269.47, 444.08) | 398.46  (338.61, 486.52) | 280.03  (225.62, 345.55) | **<0.001** |
| Iron Fe, mg | 8.72  (7.05, 10.95) | 9.00  (7.12, 11.70) | 9.00  (6.89, 10.64) | 0.264 |
| Vitamin A, retinol equivalents, µg^5^ | 210.69  (133.09, 338.84) | 231.57  (150.96, 351.08) | 171.65  (109.01, 294.53) | **0.012** |
| Vitamin D, µg | 1.71  (0.73, 2.68) | 1.82  (0.96, 3.00) | 1.33  (0.27, 2.54) | 0.069 |
| Vitamin C, mg | 35.71  (16.49, 66.60) | 41.86  (19.05, 75.89) | 27.71  (14.23, 59.51) | **0.049** |

^1^Median and interquartile range; ^2^Rural-urban differences from Mann-Whitney U tests, bold type = statistically significant (p<0.05); ^3^Megajoules; ^4^Non-starch polysaccharides; ^5^1 retinol equivalent = 1 μg of retinol or 6 μg of β-carotene**.**
