# Supplementary material for: Food consumption and nutrient intake among adolescent girls in The Gambia: a cross-sectional study with urban-rural and nutritional status comparisons
Source: BMC Nutr. 2026 Jul 17;12:215. doi: 10.1186/s40795-026-01430-0 (PMC13656378; doi:10.1186/s40795-026-01430-0)
Supplement: Supplementary file 6 [file 40795_2026_1430_MOESM6_ESM.docx]

**Supplementary file 6. Comparison of energy and nutrient intake by nutritional status (weight status and stunting categories), N=203**

|  | Median^1^  (Interquartile range) | | | | | | | |
| --- | --- | --- | --- | --- | --- | --- | --- | --- |
|  | **All** | **Underweight** | **Normal weight** | **Overweight/ obese** | **p-value^2^** | **Stunted** | **Not stunted** | **p-value^3^** |
| Energy, MJ^4^ | 9  (7, 11) | 10  (9, 12) | 9  (7, 11) | 8  (6, 10) | **0.015** | 9  (5, 18) | 9  (4, 20) | 0.635 |
| Protein, g | 57  (43, 66) | 54  (43, 66) | 53  (43, 66) | 54  (40, 67) | 0.822 | 54  (10, 89) | 53  (10, 100) | 0.531 |
| Protein, % energy | 10  (8, 12) | 10  (7, 12) | 10  (8, 11) | 10  (7, 12) | 0.666 | 10  (7, 12) | 10  (8, 12) | 0.499 |
| Total fat, g | 105  (73, 135) | 117  (73, 146) | 105  (73, 133) | 93  (58, 120) | 0.494 | 101  (33, 590) | 107  (30, 310) | 0.990 |
| Total fat, % energy | 43  (34, 51) | 42  (34, 51) | 44  (35, 51) | 36  (30, 51) | 0.631 | 42  (34, 51) | 44  (35, 51) | 0.597 |
| Saturated fat, g | 15  (8, 24) | 15  (7, 27) | 15  (8, 23) | 19  (13, 23) | 0.355 | 13  (3, 62) | 16  (3, 113) | **0.023** |
| Saturated fat, % energy | 6  (3.10) | 7  (3, 11) | 6  (3, 10) | 8  (5, 11) | 0.369 | 6  (2, 9) | 6  (4, 11) | **0.019** |
| Polyunsaturated fat, g | 5  (3, 7) | 5  (3, 7) | 5  (4, 6) | 5  (4, 6) | 0.656 | 4  (1, 14) | 5  (1, 22) | 0.096 |
| Polyunsaturated fat, % energy | 2  (1, 3) | 2  (1, 3) | 2  (2, 3) | 2  (10, 13) | 0.607 | 2  (1, 3) | 2  (2, 3) | 0.085 |
| Carbohydrate, g | 247  (211, 309) | 249  (209, 312) | 243  (208, 302) | 245  (221, 318) | 0.795 | 246  (141, 562) | 240  (126, 512) | 0.483 |
| Carbohydrate, % energy | 46  (40, 55) | 47  (39, 54) | 46  (40, 53) | 53  (40, 61) | 0.557 | 48  (40, 55) | 46  (40, 53) | 0.409 |
| Total free sugars, g | 23  (14, 36) | 27  (18, 38) | 22  (12, 34) | 24  (11, 32) | 0.144 | 22  (9. 36) | 23  (15, 35) | 0.546 |
| Total free sugars, % energy | 4  (3, 7) | 5  (3, 7) | 4  (2, 6) | 4  (2, 6) | 0.178 | 4  (2. 6) | 4  (3, 7) | 0.343 |
| Dietary fibre, NSP (Englyst), g^5^ | 17  (13, 20) | 17  (13, 21) | 16  (13, 20) | 19  (15, 22) | 0.437 | 16  (7, 41) | 16  (7, 43) | 0.811 |
| Calcium, mg | 358  (280, 451) | 353  (284, 467) | 355  (270, 442) | 340  (262, 480) | 0.865 | 325  (150, 806) | 367  (107, 1205) | **0.018** |
| Iron Fe, mg | 9  (7, 11) | 10  (8, 12) | 9  (7, 11) | 8  (6, 12) | 0.548 | 9  (4,183) | 9  (3, 32) | 0.409 |
| Vitamin A, retinol equivalents, µg^6^ | 204  (120, 334) | 199  (109, 309) | 214  (137, 352) | 276  (151, 330) | 0.179 | 184  (35, 223) | 220  (28, 313) | 0.102 |
| Vitamin D, µg | 2  (1, 3) | 2  (0, 4) | 2  (1, 3) | 2  (2, 3) | 0.835 | 2  (0, 14) | 2  (0, 15) | 0.137 |
| Vitamin C, mg | 34  (17, 66) | 35  (15, 66) | 34  (17, 66) | 44  (17, 92) | 0.837 | 26  (2, 377) | 41  (2, 102) | 0.056 |

^1^Median and interquartile range unless otherwise stated. ^2^from Kruskal-Wallis tests, bold type = statistically significant (p<0.05). ^3^from Mann-Whitney U tests, bold type = statistically significant (p<0.05); ^4^Megajoules; ^5^Non-starch polysaccharides; ^6^1 eetinol equivalent = 1 μg of retinol or 6 μg of β-carotene.
