# Supplementary material for: Food consumption and nutrient intake among adolescent girls in The Gambia: a cross-sectional study with urban-rural and nutritional status comparisons
Source: BMC Nutr. 2026 Jul 17;12:215. doi: 10.1186/s40795-026-01430-0 (PMC13656378; doi:10.1186/s40795-026-01430-0)
Supplement: Supplementary file 7 [file 40795_2026_1430_MOESM7_ESM.docx]

**Supplementary file 7.**

**(i). Food consumption (all food groups) and percentage contribution to energy and macronutrient intake**

|  | Intake per head per day and percentage contribution* of each food group to energy and macronutrients component | | | | | | | | | | | | | |
| --- | --- | --- | --- | --- | --- | --- | --- | --- | --- | --- | --- | --- | --- | --- |
| **Food group** | **Quantity consumed (g)** | **%** | **Energy (kcal)** | **%** | **Carbohydrates (g)** | **%** | **Sugars (g)** | **%** | **Protein (g)** | **%** | **Fat (g)** | **%** | **Fibre (g)** | **%** |
| Meat & Meat products | 49.60 | 3.4 | 64.75 | 2.1 | 0.07 | 0.0 | 0.04 | 0.1 | 9.44 | **12.0** | 2.97 | 2.7 | 0.01 | 0.1 |
| Fish & fish products | 54.02 | 3.7 | 105.15 | 3.4 | 0.35 | 0.1 | 0.04 | 0.0 | 10.24 | **13.1** | 6.96 | 6.3 | 0.10 | 0.5 |
| Eggs | 13.94 | 1.0 | 19.80 | 0.6 | 0.06 | 0.0 | 0.00 | 0.0 | 1.88 | 2.4 | 1.35 | 1.2 | 0.00 | 0.0 |
| Milk & cream | 23.94 | 1.7 | 28.44 | 0.9 | 1.29 | 0.3 | 1.19 | 2.8 | 0.79 | 1.0 | 2.23 | 2.0 | 0.01 | 0.1 |
| Beans, Peas & Lentils | 9.12 | 0.6 | 27.20 | 0.9 | 1.92 | 0.4 | 0.10 | 0.2 | 2.28 | 2.9 | 1.17 | 1.1 | 0.43 | 2.0 |
| Nuts & Seeds | 31.85 | 2.2 | 183.78 | 6.0 | 2.88 | 0.7 | 0.18 | 0.4 | 7.39 | 9.4 | 15.93 | **14.5** | 2.60 | **12.3** |
| Bread, flours, & crackers | 96.97 | 6.7 | 262.39 | 8.5 | 51.77 | **11.7** | 2.47 | 5.7 | 8.95 | **11.4** | 2.17 | 2.0 | 3.15 | **14.8** |
| Rice & other grains | 446.74 | **30.7** | 1435.78 | **46.7** | 319.41 | **72.2** | 0.26 | 0.6 | 29.92 | **38.2** | 4.27 | 3.9 | 6.88 | **32.5** |
| Fruit/ dried fruit | 52.16 | 3.6 | 29.48 | 1.0 | 6.71 | 1.5 | 5.29 | **12.2** | 0.44 | 0.6 | 0.17 | 0.2 | 0.68 | 3.2 |
| Vegetables/ vegetable dishes | 130.85 | 9.0 | 40.20 | 1.3 | 5.98 | 1.4 | 2.63 | 6.1 | 2.35 | 3.0 | 0.82 | 0.8 | 2.99 | **14.1** |
| Roots, tubers & bulbs | 89.32 | 6.1 | 63.17 | 2.1 | 13.40 | 3.0 | 3.82 | 8.8 | 1.15 | 1.5 | 0.61 | 0.6 | 1.78 | 8.4 |
| Oils & spreading fats | 70.76 | 4.9 | 589.84 | **19.2** | 0.93 | 0.2 | 0.31 | 0.7 | 0.30 | 0.4 | 64.99 | **59.0** | 0.33 | 1.6 |
| Cereals & Muesli | 2.28 | 0.2 | 3.79 | 0.1 | 0.67 | 0.2 | 0.18 | 0.4 | 0.12 | 0.2 | 0.06 | 0.1 | 0.04 | 0.2 |
| Pasta, pastry & doughs | 13.30 | 0.9 | 21.70 | 0.7 | 4.16 | 0.9 | 0.17 | 0.4 | 0.76 | 1.0 | 0.23 | 0.2 | 0.32 | 1.5 |
| Savoury snacks | 4.67 | 0.3 | 13.26 | 0.4 | 1.87 | 0.4 | 0.14 | 0.3 | 0.35 | 0.4 | 0.49 | 0.4 | 0.18 | 0.9 |
| Sugar & sugary snacks and puddings | 26.30 | 1.8 | 104.88 | 3.4 | 22.29 | 5.0 | 21.36 | **49.4** | 0.73 | 0.9 | 1.42 | 1.3 | 0.18 | 0.9 |
| Salt and other condiments | 20.37 | 1.4 | 50.60 | 1.7 | 3.17 | 0.7 | 0.86 | 2.0 | 0.67 | 0.9 | 3.88 | 3.5 | 0.87 | 4.1 |
| Sweetened drinks | 58.11 | 4.0 | 21.24 | 0.7 | 5.05 | 1.1 | 3.77 | 8.7 | 0.26 | 0.3 | 0.03 | 0.0 | 0.64 | 3.0 |
| Tea and coffee | 143.49 | 9.9 | 6.42 | 0.2 | 0.44 | 0.1 | 0.41 | 1.0 | 0.40 | 0.5 | 0.34 | 0.3 | 0.00 | 0.0 |

**(ii) Food consumption (all food groups) and percentage contribution to saturated and polyunsaturated fatty acids**

|  | Intake per head per day and percentage contribution* of each food group to saturated and polyunsaturated fatty acid | | | |
| --- | --- | --- | --- | --- |
| **Food group** | **Saturated fatty acid (g):** | **%** | **Polyunsaturated fatty acid (g)** | **%** |
| Meat & Meat products | 0.98 | 5.3 | 0.47 | 8.1 |
| Fish & fish products | 1.48 | 8.0 | 0.67 | **11.5** |
| Eggs | 0.38 | 2.0 | 0.21 | 3.6 |
| Milk & cream | 1.38 | 7.5 | 0.06 | 1.1 |
| Beans, Peas & Lentils | 0.02 | 0.1 | 0.02 | 0.4 |
| Nuts & Seeds | 0.27 | 1.5 | 0.39 | 6.7 |
| Bread, flours, & crackers | 0.41 | 2.2 | 0.86 | **14.8** |
| Rice & other grains | 0.89 | 4.8 | 1.40 | **24.1** |
| Fruit/ dried fruit | 0.05 | 0.3 | 0.03 | 0.4 |
| Vegetables/ vegetable dishes | 0.11 | 0.6 | 0.22 | 3.8 |
| Roots, tubers & bulbs | 0.20 | 1.1 | 0.08 | 1.4 |
| Oils & spreading fats | 10.95 | **59.3** | 0.15 | 2.6 |
| Cereals & Muesli | 0.03 | 0.2 | 0.01 | 0.2 |
| Pasta, pastry & doughs | 0.06 | 0.3 | 0.07 | 1.2 |
| Savoury snacks | 0.02 | 0.1 | 0.03 | 0.5 |
| Sugar & sugary snacks and puddings | 0.62 | 3.4 | 0.15 | 2.6 |
| Salt and other condiments | 0.37 | 2.0 | 0.98 | **16.8** |
| Sweetened drinks | 0.01 | 0.0 | 0.00 | 0.1 |
| Tea and coffee | 0.26 | 1.4 | 0.00 | 0.0 |

**(iii) Food consumption (all food groups) and percentage contribution to selected micronutrients**

|  | Intake per head per day and percentage contribution^1^ of selected micronutrients | | | | | | | | |  |
| --- | --- | --- | --- | --- | --- | --- | --- | --- | --- | --- |
| **Food group** | **Vitamin A (Retinol equivalents; µg)** | **%** | **Vitamin D (µg):** | **%** | **Vitamin C (mg)** | **%** | **Calcium (mg)** | **%** | **Iron (mg)** | **%** |
| Meat & Meat products | 47.60 | **15.2** | 0.14 | 6.2 | 0.13 | 0.2 | 4.08 | 0.9 | 0.98 | 9.2 |
| Fish & fish products | 5.53 | 1.8 | 1.57 | **69.8** | 0.02 | 0.0 | 20.47 | 4.6 | 0.25 | 2.4 |
| Eggs | 16.82 | 5.4 | 0.42 | **18.8** | 0.00 | 0.0 | 7.25 | 1.6 | 0.26 | 2.5 |
| Milk & cream | 20.95 | 6.7 | 0.03 | 1.3 | 0.49 | 0.7 | 32.46 | 7.3 | 0.05 | 0.5 |
| Beans, Peas & Lentils | 0.16 | 0.1 | 0.00 | 0.0 | 0.00 | 0.0 | 18.47 | 4.2 | 0.28 | 2.6 |
| Nuts & Seeds | 0.00 | 0.0 | 0.00 | 0.0 | 0.01 | 0.0 | 1.56 | 0.4 | 1.01 | 9.5 |
| Bread, flours, & crackers | 0.00 | 0.0 | 0.00 | 0.0 | 0.00 | 0.0 | 120.66 | **27.2** | 1.69 | **15.9** |
| Rice & other grains | 0.00 | 0.0 | 0.00 | 0.0 | 0.00 | 0.0 | 101.34 | **22.9** | 1.50 | **14.1** |
| Fruit/ dried fruit | 31.49 | **10.1** | 0.00 | 0.0 | 34.87 | **53.1** | 14.72 | 3.3 | 0.35 | 3.2 |
| Vegetables/ vegetable dishes | 89.94 | **28.7** | 0.00 | 0.1 | 11.85 | **18.1** | 50.02 | **11.3** | 1.41 | **13.2** |
| Roots, tubers & bulbs | 37.11 | **11.9** | 0.00 | 0.0 | 5.32 | 8.1 | 20.86 | 4.7 | 0.34 | 3.2 |
| Oils & spreading fats | 40.76 | **13.0** | 0.04 | 1.6 | 0.00 | 0.0 | 0.70 | 0.2 | 0.00 | 0.0 |
| Cereals & Muesli | 0.50 | 0.2 | 0.03 | 1.1 | 0.01 | 0.0 | 4.54 | 1.0 | 0.08 | 0.7 |
| Pasta, pastry & doughs | 0.70 | 0.2 | 0.01 | 0.4 | 0.01 | 0.0 | 5.90 | 1.3 | 0.12 | 1.1 |
| Savoury snacks | 0.02 | 0.0 | 0.00 | 0.0 | 0.08 | 0.1 | 1.02 | 0.2 | 0.05 | 0.5 |
| Sugar & sugary snacks and puddings | 4.36 | 1.4 | 0.01 | 0.6 | 0.02 | 0.0 | 10.33 | 2.3 | 0.12 | 1.2 |
| Salt and other condiments | 5.56 | 1.8 | 0.00 | 0.0 | 0.85 | 1.3 | 6.37 | 1.4 | 1.89 | **17.7** |
| Sweetened drinks | 6.40 | 2.1 | 0.00 | 0.1 | 11.96 | **18.2** | 11.34 | 2.6 | 0.27 | 2.5 |
| Tea and coffee | 5.11 | 1.6 | 0.00 | 0.0 | 0.00 | 0.0 | 11.18 | 2.5 | 0.00 | 0.0 |

^1^Contributions of 10% or more are highlighted in bold
